
JOURNAL INFORMATION
==============================
NLM Title Abbreviation: Innov Aging
Journal ID: Innov Aging
NLM Journal ID: 101703706
Journal ID: innovateage

Innovation in Aging

EISSN: 2399-5300
Publisher: Oxford University Press

ARTICLE INFORMATION
==============================
PMCID: PMC7742696
DOI: 10.1093/geroni/igaa057.3132
Article ID: igaa057.3132
Article version: 1
Subjects: Abstracts, Session 7695 (Symposium), AcademicSubjects/SOC02600

Public Policy Advisory Committee Perspective

Harootyan Linda
Harootyan, LLC , Wilmington, North Carolina, United States
Publication date: 2020
Electronic publication date: 2020 Dec 16
Volume: 4
Issue: Suppl 1
Issue title: Program Abstracts from The GSA 2020 Annual Scientific Meeting “Turning 75: Why Age Matters”
First page: 853
Last page: 853
Copyright: © The Author(s) 2020. Published by Oxford University Press on behalf of The Gerontological Society of America.
Copyright year: 2020
License: This is an Open Access article distributed under the terms of the Creative Commons Attribution License (http://creativecommons.org/licenses/by/4.0/), which permits unrestricted reuse, distribution, and reproduction in any medium, provided the original work is properly cited.
License URL: https://creativecommons.org/licenses/by/4.0/

Page count: 1

==============================
### PUBLIC POLICY ADVISORY COMMITTEE PERSPECTIVE

Linda Harootyan, *Harootyan*
*2. , LLC, Wilmington, North Carolina, United States*
